# Supplementary material for: BARS Influences Neuronal Development by Regulation of Post-Golgi Trafficking
Source: Cells. 2022 Apr 13;11(8):1320. doi: 10.3390/cells11081320 (PMC9026897; doi:10.3390/cells11081320)
Supplement: Supplementary file 1 [file cells-11-01320-s001.zip › cells-1580734-supplementary.pdf]

Supplementary Information

Bars Influences Neuronal Development by Regulation of Post-Golgi Trafficking

Supplementary Figure S1: shRNA-BARS-GFP transfection reduces BARS expression in cultured cells.

Supplementary Figure S2: Schematic representation of BARS domain structure and BARS mutants cDNA constructs used in this paper.

Supplementary Figure S3: BARS do not regulate proliferation, differentiation and centrosome orientation in cortical neurons.

Supplementary Figure S4: Regulated secretion/aggregation FM based system

Supplementary Figure S5: Distribution of ApoER2-GFP in cultured neurons.

Supplementary Figure S6: BARS regulates trafficking of L1.

Supplementary Figure S7: BARS does not regulate trafficking of P75NTR.

Supplementary Figure S1

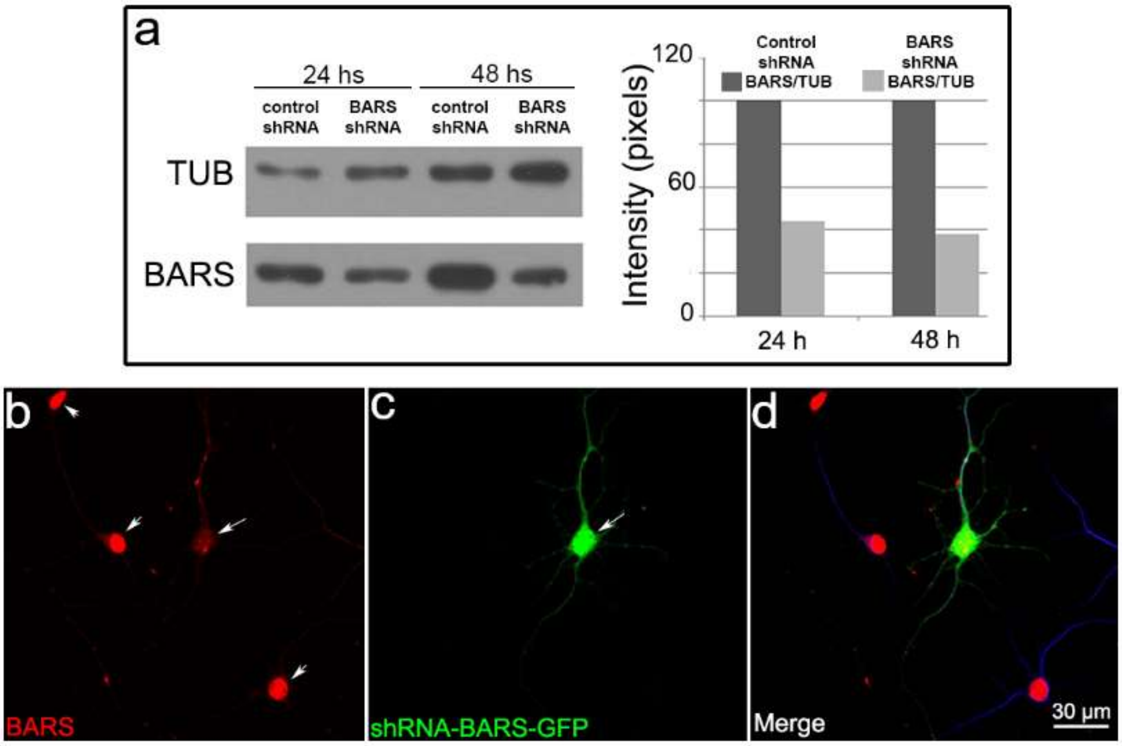

Figure S1. shRNA-BARS-GFP transfection reduces BARS expression in cultured cells. (**a**) (left) Western-blot showing levels of BARS protein obtained from B16 cell extracts co-transfected with control scramble sc-shRNA-BARS-GFP (control shRNA) or shRNA-BARS-GFP (BARS shRNA) plasmids for 24 hs and 48 hs. 20 μg of total cellular protein were loaded in each lane. The blot was revealed with the polyclonal antibody anti-BARS. (right) Densitometric analysis of Western blots from control- and BARS shRNA-treated cells. 3 blots were analyzed for each experimental condition. Note the significant decrease in BARS protein levels after transfection with the BARS shRNA. (**b**-**d**) Confocal images showing BARS (red) staining transfected with the shRNA-BARS-GFP (green) in 7 DIV hippocampal cultured neurons stained with MAP2 (blue in d). Note that neurons expressing the shRNA (arrows) show reduced levels of BARS immunofluorescence compared to non-transfected cells (arrowheads).

Supplementary Figure S2

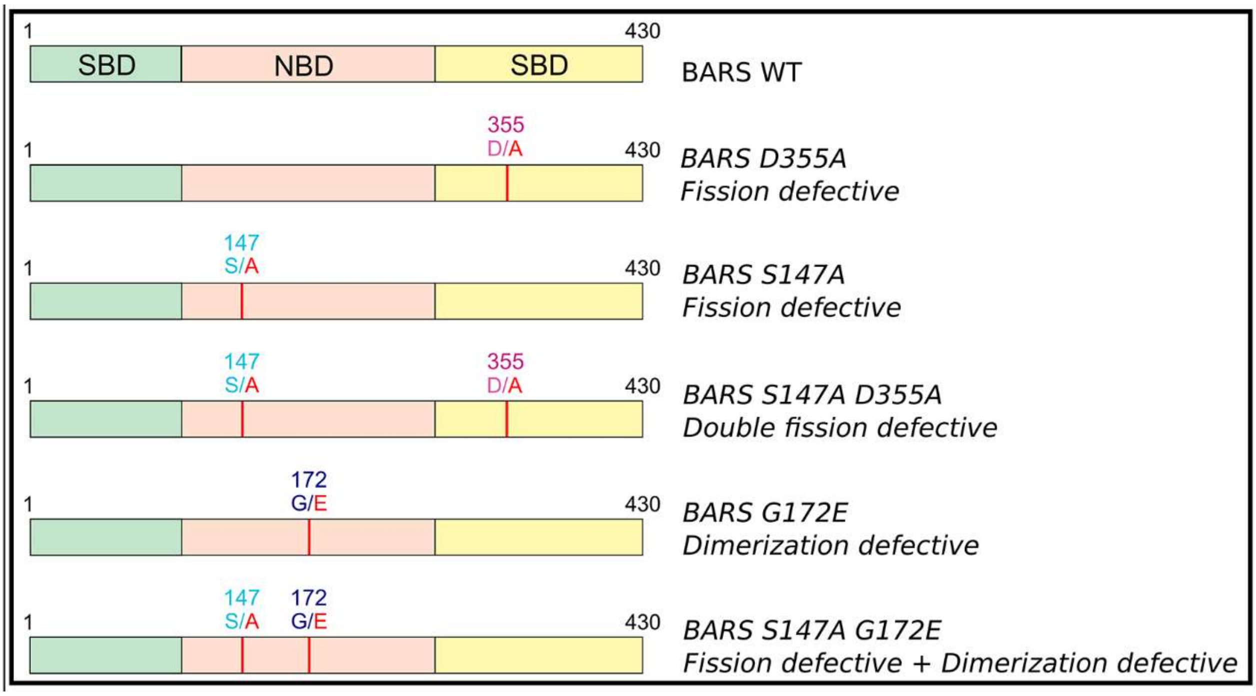

Figure S2. Schematic representation of BARS domain structure and BARS mutants cDNA constructs used in this paper. NBD: Nucleotide binding domain. SBD: Substrate binding domain.

Supplementary Figure S3

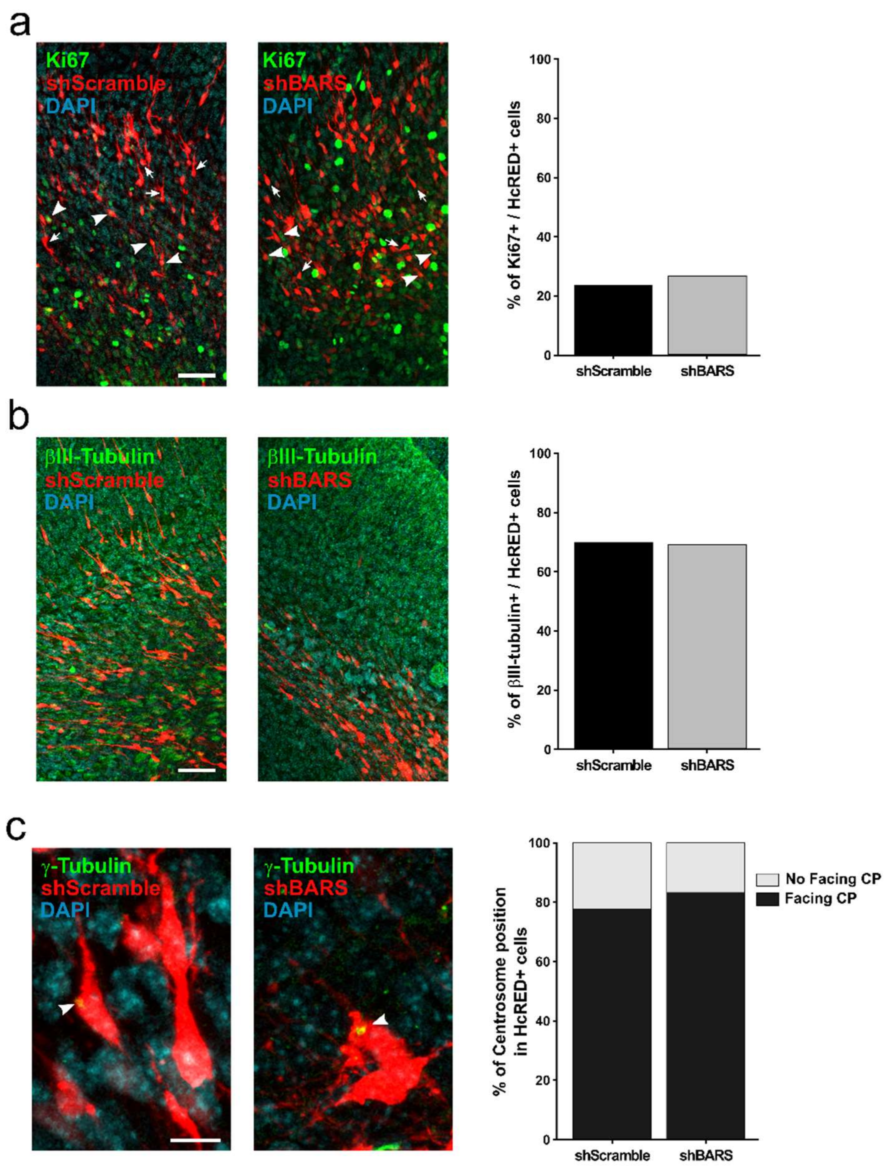

Figure S3. BARS do not regulate proliferation, differentiation and centrosome orientation in cortical neurons. (a) Left and middle panels: Representatives images of coronal cortical slices of mouse brain (embryonic day E18.5), expressing sc-shRNA-BARS-HcRED (shScramble) or shRNA-BARS-HcRED (shBARS) respectively after IUE and stained with a polyclonal Ab against the proliferation marker Ki67 and DAPI. Arrow point to cells positive for HcRED but negative for Ki67. Arrowheads point to cells positive for HcRED and Ki67. Right panel: Quantification of the percentage of HcRED positive and Ki67 positive shScramble (23.3%) and shBARS (26.5%) electroporated neurons in. Scale bar: 50 µm. (b)

Left and middle panels: Representatives images of coronal cortical slices of mouse brain (embryonic day E18.5), expressing sc-shRNA-BARS-HcRED (shScramble) or shRNA-BARS-HcRed (shBARS) respectively after IUE and stained with a mAb against the early neuronal marker βIII-tubulin and DAPI. Scale Bar: 50 μm. Right panel: Quantification of the percentage of HcRED positive and βIII-tubulin positive shScramble (70.1%) and shBARS (69.3%) electroporated neurons. Scale bar: (c) Left and middle panels: Representatives images showing the distribution of centrosome in a cortical neuron of mouse brain (embryonic day E18.5) transfected by IUE with sc-shRNA BARS-HcRED (scScramble) or shRNA-BARS-HcRed (shBARS) respectively stained with γ-Tubulin and DAPI. Scale Bar: 50 μm. Right panel: Quantification of centrosome position in shScramble (77,8 % facing cortical plate) and shBARS (83,3% facing cortical plate) expressing neurons at radial migration zone, RMZ.

Supplementary Figure S4

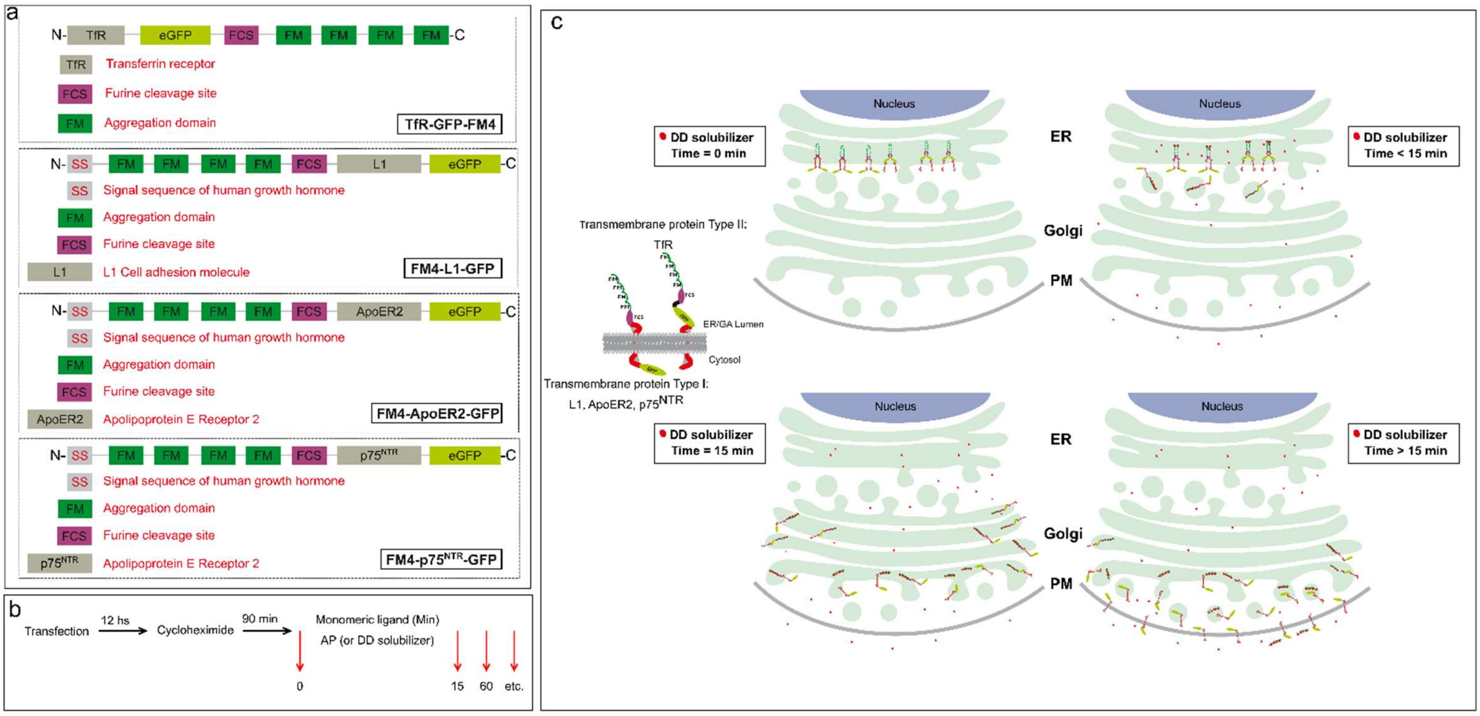

Figure S4. Regulated secretion/aggregation FM based system. (**a**) Schematic representation of type II (TfR-GFP-FM4) and type I (FM4-L1-GFP, FM4-ApoER2-GFP and FM4-p75^{NTR}-GFP) plasma membrane protein FM4 plasmids used in this paper. (**b**) Schematic representation of the regulated secretion/aggregation FM based protocol in hippocampal neuronal culture. (c, left) Schematic diagram showing the structure of the FM4-GFP type I and Type 2 plasma membrane proteins (c, right) Schematic drawings illustrating the predicted expression pattern of FM-GFP plasma membrane protein. In the absence of DD solubilizer, transmembrane proteins (red) fused to FM domains (green) and to GFP (light green) multimerize causing clustering and retentions of the fusion protein within the ER. When DD solubilizer is present in the medium bind the FM4 domain causing disaggregation of the expressed transmembrane fusion protein, releasing it from the ER and allowing it to move through the secretory pathway.

Supplementary Figure S5

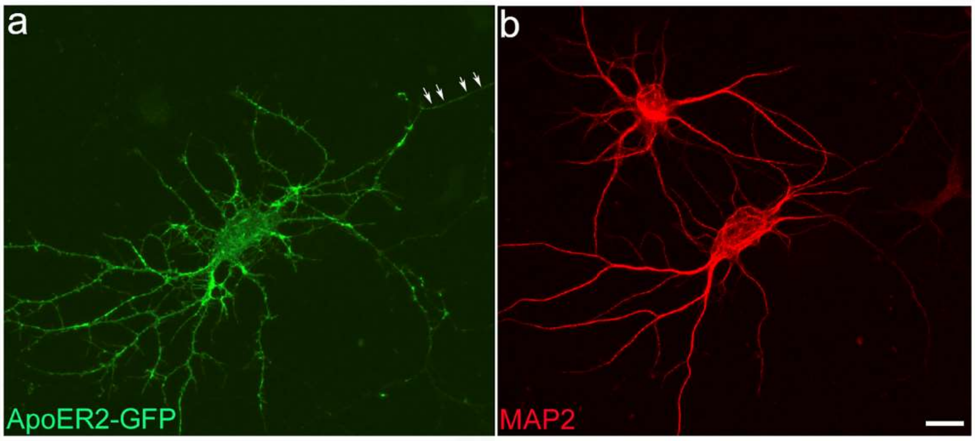

Figure S5. Distribution of ApoER2-GFP in cultured neurons. Confocal images showing the distribution of ectopically expressed ApoER2-GFP (left) and MAP2 (right, red) in a 7 DIV hippocampal neuron. Note that the distribution of ApoER2-GFP mimic that of MAP2. Scale bar: 10 μm.

Supplementary Figure S6

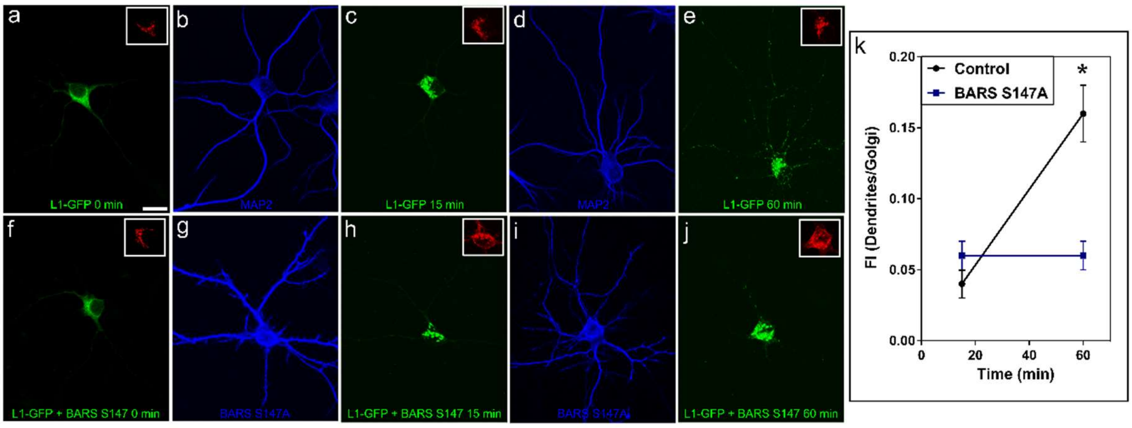

Figure S6. BARS regulates trafficking of L1. (**a-e**) A series of confocal images showing the distribution of ectopically expressed FM4-L1-GFP visualized 0, 15 and 60 min after de addition of DD solubilizer. Inset (red), distribution of ectopically expressed GalT2-mCherry. MAP2 labeling in shown in b and d (blue). (**f-j**) A series of confocal images showing the distribution of ectopically expressed FM4-L1-GFP co-transfection with BARS S147A and visualized 0, 15 and 60 min after de addition of DD solubilizer. Inset (red), distribution of ectopically expressed GalT2-mCherry. BARS S147A expression is shown in g and i (blue). Scale bar: 10 μm. (**i**) Graph showing the fluorescent intensity (FI) ratio between L1-GFP labeling in dendrites and the Golgi area 15 and 60 min after the addition of DD solubilizer in control- and BARS S147A-expressing neurons. Graphs represent mean ± S.E.M. *<0.05; Student's t-test, n = 10, 12, 9 and 10 neurons for control 15, 60 min, BARS S147A 15 and 60 min respectively pooled from at least three independent cultures.

Supplementary Figure S7

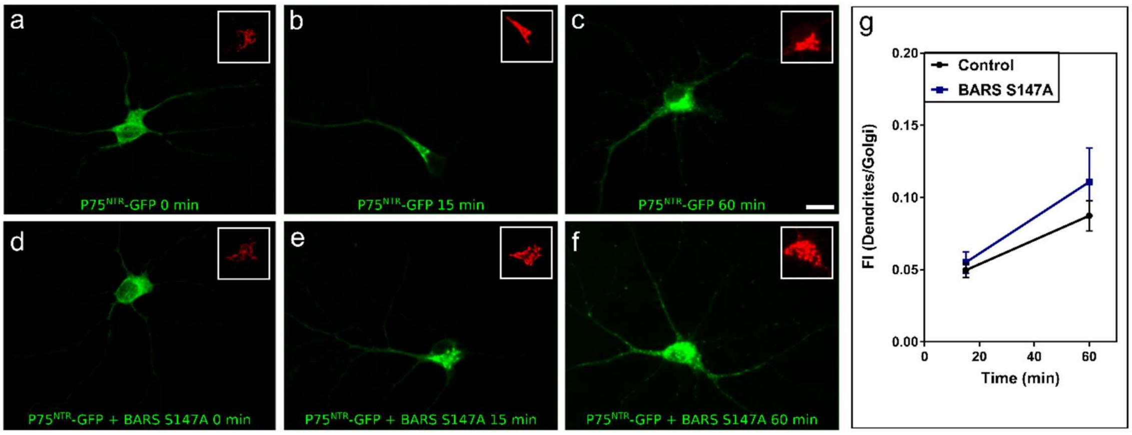

Figure S7. BARS does not regulate trafficking of P75NTR. (**a**-**f**) A series of confocal images showing the distribution of ectopically expressed FM4-P75^{NTR}-GFP alone or after co-transfection with BARS S147A and visualized 0, 15 and 60 min after de addition of DD solubilizer. Inset (red), distribution of ectopically expressed GalT2-mCherry. Scale bar: 10 µm. (**g**) Graph showing the fluorescent intensity (FI) ratio between L1-GFP labeling in dendrites and the Golgi area 15 and 60 min after the addition of DD solubilizer in control- and BARS S147A-expressing neurons. Graphs represent mean ± S.E.M. not significant; Student's t-test, n = 12, 8, 10 and 10 neurons for control 15, 60 min, BARS S147A 15 and 60 min respectively pooled from at least three independent cultures.
